# Understanding the Evidence Gaps in the Epidemiology and Burden of Lupus Nephritis and Membranous Nephropathy: A Targeted Literature Review

**DOI:** 10.36469/001c.166255

**Published:** 2026-10-07

**Authors:** Francesca Barion, Vasileios Pardalidis, Alisha Robert, Catherine Kielar

**Affiliations:** 1 argenx, Milan, Italy; 2 Avalere Health, London, UK

**Keywords:** burden, cost, epidemiology, end-stage renal disease, health-related quality of life, lupus nephritis, membranous nephropathy

## Abstract

**Background:**

Lupus nephritis (LN) and membranous nephropathy (MN) are associated with progression to chronic kidney disease and end-stage renal disease (ESRD), but evidence describing the full disease burden across countries remains limited and fragmented.

**Objectives:**

This targeted literature review (TLR) aimed to characterize the disease burden of LN and MN, including epidemiology and clinical, humanistic, and economic outcomes, and identify unmet needs and current knowledge gaps.

**Methods:**

A targeted, nonsystematic search of MEDLINE and EMBASE was conducted for English-language articles published between January 2013 and August 2023 in the United States (US), the United Kingdom, Germany, France, China, and Japan.

**Results:**

Searches identified 26 relevant articles for LN and 11 for MN. LN epidemiology studies were limited to the US; incidence was estimated at 1:100 000, with higher risk in older individuals, females, and African Americans. MN is rare, with reported annual incidences of 10 to 12 per million in North America and 2 to 17 per million in Europe. Prevalence appears to be increasing for both diseases. LN was associated with substantial progression to ESRD, with 21.1% to 36.8% of patients progressing within 5 to 10 years. ESRD represented a major economic driver, with US monthly costs of approximately $15 000 per patient with LN. MN burden data were more limited but indicated risk of severe long-term renal complications and ESRD. Humanistic burden data were sparse; reported health-related quality-of-life impacts include renal symptom domains not captured by generic instruments, indicating a need for disease-specific measures.

**Conclusions:**

Despite evidence of substantial burden, important gaps remain. Epidemiological data were geographically limited, with incidence varying widely, potentially reflecting differences in populations, treatment eras, and ethnicity. Long-term data beyond 5 to 10 years were limited, and robust syntheses evaluating prognostic factors influencing progression to ESRD were lacking. The absence of renal-specific patient-reported outcome measures and inconsistent clinical definitions further limits comparability and treatment value assessment. LN and MN are associated with significant clinical, economic, and humanistic burden, especially for patients with ESRD, but certainty and generalizability are limited by the focused TLR methods and heterogeneous evidence base. More robust data would help characterize long-term outcomes and support more informed value assessments.

## BACKGROUND

Lupus nephritis (LN) and membranous nephropathy (MN) are types of glomerulonephritis associated with a high burden due to progression to chronic kidney disease and end-stage renal disease (ESRD).^1^ LN is an autoimmune disease caused when systemic lupus erythematosus (SLE) affects parts of the kidneys, resulting in impaired kidney function.^2^ MN is a common cause of nephrotic syndrome in adults and can have various underlying causes, including autoimmune disorders, infections, use of certain medications, and exposure to certain toxins.^3^ MN is classified into primary (75%-80% of cases) and secondary (20%-25%) etiologies and is a rare disease with a highly variable prognosis.^4^ The course of the disease ranges from spontaneous remission to persistent proteinuria or ESRD, making the burden of the disease challenging to characterize.

Treatment guidelines for LN and MN provide some direction but leave uncertainties in practice. For LN, multiple treatment options are available,^5–9^ but there is a lack of clarity on the optimal timing and sequencing of treatment and the changes needed upon disease progression. Complete response rates for current therapies are typically up to approximately 40%, meaning that many patients do not achieve a complete response.^5,10^ Limited risk-based guidance is available for MN,^11^ but there are no approved/reimbursed treatments; recommended treatments are currently used off-label, and practice varies between regions.

The unmet need in both conditions is therefore defined by prognosis rather than frequency; although both are rare, a substantial proportion of patients progress to ESRD despite guideline-recommended therapy, and for MN no treatment is approved or reimbursed. Remaining needs relate to reducing the risk of ESRD and associated clinical and humanistic burden and healthcare costs. However, an understanding of the full picture regarding costs and burden globally is limited, and there are no robust registries focused on these diseases. Past uncertainty and lack of long-term data have led to access barriers for targeted therapies.^12^ An assessment of the literature is needed to confirm the information that is known vs what is missing, and the evidence needed to address these gaps. The aim of this targeted literature review (TLR) was to establish what is known regarding the disease burden of LN and MN and identify key evidence gaps and evidence-generation priorities in these conditions.

## METHODS

A TLR and synthesis of current literature on the epidemiology and the clinical, economic, and humanistic burden of LN and MN were conducted to identify relevant articles published between January 2013 and August 2023 in the United States (US), the United Kingdom (UK), Germany, France, China, and Japan. Supplementary hand searches were also conducted using Google to identify further relevant information from gray literature, nonindexed sources, and recent developments potentially not captured in the primary databases (eg, clinical guidelines, randomized controlled trials related to standard of care, and health technology assessment [HTA] outcomes).

The TLR used a focused, pragmatic search with predefined restrictions and, unlike a systematic literature review (SLR), was not intended to be exhaustive. A disease area review (DAR) was defined as a broad narrative or expert-authored synthesis of a disease and its burden, which does not necessarily include a fully reproducible search process or formal appraisal. Two DARs were included as sources; findings attributed to them are secondary rather than primary data.

The 6 countries were selected pragmatically to span North American, European, and Asian systems where formal value assessment frameworks are commonly required, rather than according to disease or economic burden.

### Search Strategy

Relevant studies were identified by searching the MEDLINE and EMBASE electronic databases. A snowballing technique was used to search relevant SLRs and DARs. Search terms used included LN, MN, prevalence/incidence, mortality/morbidity/hospitalization, direct/indirect costs, and quality of life (QoL).

Database-specific strategies combined controlled vocabulary (Medical Subject Headings for MEDLINE; Emtree for EMBASE) with free-text terms adapted to each interface. The full search strategies are summarized in **Supplementary Tables S1 and S2**. Records were combined and duplicates removed electronically by matching title, first author, journal, and year, with residual duplicates identified during screening.

Hand searching covered Google and Google Scholar for clinical guidelines, HTA appraisal documents, and conference and other nonindexed reports. No additional eligible primary burden studies were identified; guidelines and HTA materials are cited for context only.

### Data Review and Extraction

The identified publications were first screened by 2 reviewers at the title/abstract level based on the predefined study eligibility criteria (**Supplementary Table S1**). Any discrepancies were discussed and resolved. The identified publications were then screened at the full-text level. Relevant data were extracted into a data extraction template in Excel. Data fields for extraction included author, year, study design, setting, sample size, disease severity, and the following research question-specific endpoints for LN and MN:

- Research question 1 (epidemiology): prevalence, incidence
- Research question 2 (clinical): mortality, morbidity, hospitalization, ESRD
- Research question 3 (humanistic): physical symptoms, emotional impact, QoL
- Research question 4 (economic): direct and indirect costs

Data were extracted by 1 reviewer and checked by a second, with discrepancies resolved by discussion. Consistent with the targeted design, no formal risk-of-bias or quality appraisal and no quantitative synthesis were undertaken; findings are reported descriptively.

## RESULTS

Of the initial 1994 records identified for LN, 26 met the TLR criteria (**Supplementary Figure S1a**). Most of the included articles reported data on clinical burden (n = 19), 5 reported epidemiological data, 3 reported humanistic burden, and 4 reported economic burden. Characteristics of the included studies for LN are presented in **Table 1** and **Supplementary Tables S3-S6**.

Of the 480 records identified for MN, 11 met the TLR criteria (**Supplementary Figure S1b**). Most of these reported epidemiological data (n = 9), 7 reported data on clinical burden, 1 (a DAR) reported humanistic burden, and 1 reported data on economic burden. Only 1 article included epidemiology data on pediatric patients (aged <14 years). Characteristics of the included studies for MN epidemiology and clinical and economic burden are presented in **Table 1** and **Supplementary Tables S7-S9**.

**Table 1.** Characteristics of Identified Studies for Lupus Nephritis and Membranous Nephropathy

**Author (Year)**	**Country**	**Study Type**	**Sample Size (No. of Centers or Data Source as Applicable)**	**Key Findings**
**Lupus Nephritis**				
Hocaoglu (2023)^13^	US	Retrospective cohort study	72 (LUMEN registry)	Epidemiology Incidence: 1.3:100 000 (average, 1976-2018) Prevalence: 21.2:100 000 (2015). While the overall incidence of LN has remained relatively constant (1976-1989, 0.7:100 000; 2000-2018, 1.3:100 000), prevalence has increased from 16.8:100 000 (in 1985) to 21.2:100 000 (in 2015) Sex: overall incidence rate of 1.5:100 000 among females and 0.5:100 000 among males (1976-2018)
				Clinical ESRD: 10% (5-year), 13% (10-year) Survival: 89% (5-year), 70% (10-year)
Bell (2022)^14^	US	Retrospective cohort study	2916 (Optum)	Epidemiology: 25%^a^ of patients with SLE and LN
				Clinical: HCRU Inpatient stays: 1195 (41%) Outpatient visits: 2916 (100%) ER visits: 1686 (57.8%) Pharmacy claims: 2723 (93.4%)
				Economic: Direct costs (mean total annual health care cost per flare) LN + any severity of a flare: $5842 LN + mild flare: $2753 LN + moderate flare: $4561 LN + severe flare: $29 148
Dall’Era (2023)^15^	US	Retrospective cohort study	21 251 (Optum)	Epidemiology Incident cases: 53.6% Prevalent cases: 46.5% 37.9% of patients with SLE and LN
				Clinical: ESRD, 10.5% (mean follow-up, 30.6 months)
				Economic: All-cause monthly medical costs Low disease activity: $2523 Active disease: $4777 ESRD: $18 084
Tedeschi (2019)^16^	US	Retrospective cohort study	9449 (Medicaid)	Epidemiology: 19.8% of patients with SLE and LN
Feldman (2013)^17^	US	Retrospective cohort study	7388 (Medicaid)	Epidemiology Average incidence rate: 6.85 per 100 000 person-years (2002-2004) Prevalence: 30.9 per 100 000 (2000-2004); 21.5% of patients with SLE and LN Age: Incidence 8.60 vs 5.14 per 100 000 person-years in patients aged 50-64 years vs 18-29 years, respectively Sex: Incidence 8.98 vs 1.47 per 100 000 person-years in females vs males, respectively Race: Incidence 10.65 for African Americans vs 4.07 for white populations, per 100 000 person-years
Khosroshahi (2023)^18^	US	Retrospective cohort study	173 (5 centers)	Clinical ESRD: 23.1% (3-year) Mortality: 6.1% (3-year)
Duong (2023)^19^	US	Retrospective cohort study	125 (single)	Clinical: ESRD, 45.7% (5-year)
Tian (2022)^20^	China	Retrospective cohort study	1264 (single)	Clinical: Survival (5-year, 10-year) Early-onset LN: 67.6%, 50.5% Late-onset LN: 89.8%, 84.6%
Gisca (2021)^21^	UK	Retrospective cohort study	219 (single)	Clinical ESRD: 28.9% (5-year), 36.8% (10-year) Mortality (5-year): 19%
Farinha (2020)^22^	UK	Retrospective cohort study	LN Class III + IV: 135 LN Class V: 38 (single)	Clinical: ESRD (median follow-up, 12 years) LN Class III + IV: 25% LN Class V: 8% LN Class III/IV + V: 14% Renal survival (5-, 10-, 15-, 20-year, cumulative): 94%, 86%, 80%, 76%
Chen (2020)^23^	China	Retrospective cohort study	LN + no AKI: 82 LN + AKI 1: 40 LN + AKI 2: 26 LN + AKI 3: 19 (single)	Clinical: 10-year survival LN + no AKI: 94.5% LN + AKI 1: 81.8% LN + AKI 2: 44.9% LN + AKI 3: 14.6%
Ntatsaki (2018)^24^	UK	Retrospective cohort study	40 (2 centers)	Clinical ESRD (42-year): 33.5% Survival: 95% (5-year), 80% (8.6-year)
Feldman (2018)^25^	US	Retrospective cohort study	LN + female: 2467 LN + male: 283 (MAX)	Clinical: ESRD (5-year): LN + female: 21.1% LN + male: 22.3% HCRU Female: Inpatient stays: 254, outpatient stays: 1829, ER visits: 952 Male: Inpatient stays: 44, outpatient stays: 134, ER visits: 60
Pruthi (2016)^26^	UK	Retrospective cohort study	190 (multicenter)	Clinical: 10-year survival: 88%
Feldman (2015)^27^	US	Retrospective cohort study	7133 (MAX)	Clinical: HCRU Inpatient stays: 1825
Gomez-Puerta (2015)^28^	US	Retrospective cohort study	12 533 (USRDS)	Clinical: Survival (1-, 5-, 10-year): African American: 88%, 63%, 49% Whites: 87%, 67%, 57% Asians: 95%, 81%, 67% Native Americans: 89%, 66%, 46%
Sexton (2015)^29^	US	Retrospective cohort study	16 650 (single)	Clinical: ESRD (5-year): 33%
Furst (2013)^30^	US	Retrospective cohort study	907 (MCO claims)	Clinical: HCRU Inpatient stays: 438 (48.2%)
				Economic: Direct costs (mean total healthcare annual cost per patient): $33 472
Rong (2022)^31^	China	Retrospective cohort study	1406 (single)	Clinical ESRD (median follow-up, 52 months): LN + normotensive, 5.5%; LN + hypertensive, 5.1% Survival (5-, 10-, 15-year): LN + normotensive: 91.2%, 86.3%, 81.3%; LN + hypertensive: 83%, 74.1%, 66.5%
Sim (2018)^32^	US	Retrospective cohort study	411 (multicenter)	Clinical: ESRD (4.5-year): 12%
Bell (2023)^33^	US	Retrospective cohort study	2310 (Optum)	Clinical: HCRU Inpatient stays: 966 (41.8%) Outpatient stays: 2085 (90.3%) ER visits: 1422 (61.6%) Pharmacy claims: 2280 (98.7%)
				Economic Direct costs (mean total all-cause annual costs per patient, including costs for inpatient stays and outpatient visits): $50 975 Mean (SD) total all-cause costs per patient in the LN cohort (50 975[86 281])werealsosignificantlyhighervstheSLEwithoutLNcohort(26 262 [52 720]), *P* < .001.
Hu (2022)^34^	China	Case-control study	50 (single)	Humanistic HADS-A: Median 7.0 (IQR: 6.0-10.0) for patients with SLE and LN; median 6 (IQR: 5.0-8.0) for those with SLE without LN HADS-D: Median 7.5 (IQR: 6.0-11.0) for patients with SLE and LN; median 6 (IQR: 5.0-8.3) for those with SLE without LN Anxiety rate: 38% vs 28% Depression rate: 50% vs 30%
Jolly (2018)^35^	Global (US, Canada, Mexico, Argentina, Turkey, the Philippines, China)	Cross-sectional study	Nonactive LN: 410 Active LN: 129 (LupusPRO)	Humanistic: According to LupusPRO, SLE patients with LN have poor HRQoL, and SLE patients with active LN have poorer HRQoL than those with nonactive LN.
Mozaffarian (2016)^36^	US	Cross-sectional study	206 (US Adelphi Lupus DSP data; No. of centers unclear)	Humanistic: Dissatisfaction with disease control was reported by 25% of patients and ~33% of nephrologists. Dissatisfaction was associated with LN severity and various signs and symptoms of disease.
**Membranous Nephropathy**				
Hu (2020)^37^	China	Cross-sectional retrospective study	8644 (single)	Epidemiology Prevalence: 2009-2013: 15.98%; 2014-2018: 30.81%, *P* = .0004 Proportion of patients with MN in study: ~25% (8644/34 630)
Pruthi (2016)^26^	UK	Cross-sectional retrospective study	183 (multicenter)	Epidemiology: Proportion of patients with MN in study: ~3.9% (183/4750)
				Clinical Survival: 154 Deaths: 29 Median follow-up to death: 6.2 years
Glenn (2020)^38^	US	Prospective, multicenter, observational cohort study	329 (72 centers)	Epidemiology Prevalence: 329 of 1741 Proportion of patients with MN in study: 19%
				Clinical: HCRU Inpatient stays: 25 ER visits: 25^b^
Ronco (2021)^39^	France and US	Disease review	NA	Epidemiology: Incidence 10-12 per million in North America 2-17 per million in Europe
Nazareth (2019)^40^	US	Retrospective observational analysis	2689 (IBM MarketScan database)	Clinical HCRU: MN HCC: Inpatient stays: 1.97 Mean inpatient LoS: 14.2 days Outpatient office visits: 5.17 Other outpatient visits: 5.91 ER visits: 1.67 Pharmacy claims: 1.67^c^ HCRU: MN NHCC: Inpatient stays: 0.31 Mean inpatient LoS: 1.4 days Outpatient office visits: 9.39 Other outpatient visits: 21.23 ER visits: 0.54 Pharmacy claims: 0.54^c^
				Economic Direct costs: MN HCC: Prescription costs: $103 382 Inpatient costs: $111 140 Outpatient costs: $187 085 Outpatient office: $2555 Outpatient ED: $1787 Outpatient other: $182 744 Outpatient hospital: $88 729 ESRD facilities: $67 302 Other facilities: $26 731 Direct costs: MN NHCC Prescription costs: $5050 Inpatient costs: $7761 Outpatient costs: $14 343 Outpatient office: $1162 Outpatient ED: $498 Outpatient other: $12 683 Outpatient hospital: $7149 ESRD facilities: $2629 Other facilities: $2905
Storrar (2022)^41^	UK	Retrospective cohort study	178 (NR)	Epidemiology: Proportion of anti-PLA2R+ patients, 59.6%^d^
				Clinical: Remission Spontaneous remission, 33.7%; complete remission, 37.6%; nephrotic proteinuria progression: 39.9%
Yin (2020)^42^	China	Retrospective cohort study	114 (single)	Epidemiology: Proportion of anti-PLA2R+ patients: PLA2R+: 71%; PLA2R−: 29%
				Clinical 12 months follow-up for SAb−/GAg−: 0% ESRD Partial remission, 10%; complete remission, 90% 12 months follow-up for SAb−/GAg+: 1% ESRD Partial remission, 47.80%; complete remission, 52.2% 12 months follow-up for SAb+/GAg+: 3% ESRD: Partial remission, 32.1%; complete remission, 58%
Deng (2022)^43^	China	Retrospective cohort study	447 (single)	Epidemiology: Proportion of anti-PLA2R+ patients PLA2RAb nonhigh titer overall: 316/447 PLA2RAb high titer overall: 131/447 PLA2RAb nonhigh titer treated with CTX: 204/275 PLA2RAb high titer treated with CTX: 71/275 PLA2RAb nonhigh titer treated with TAC: 112/172 PLA2RAb high titer treated with TAC: 60/172 Individuals with high titer antibody had significantly higher urinary protein levels and lower serum albumin levels.
				Clinical PLA2RAb nonhigh titer overall Partial remission, 44.8%; complete remission, 44.7% PLA2RAb high titer overall: Partial remission, 23.5%; complete remission, 20% PLA2RAb nonhigh titer treated with CTX Partial remission, 44.6%; complete remission, 45.2% PLA2RAb high titer treated with CTX Partial remission, 28.6%; complete remission, 27% PLA2RAb nonhigh titer treated with TAC Partial remission, 44.9%; complete remission, 43.9% PLA2RAb high titer treated with TAC Partial remission, 17.3%; complete remission, 11.5%
Sun (2022)^44^	China	Retrospective cohort study	155 (NR)	Epidemiology: Proportion of anti-PLA2R+ patients: 62%
				Clinical Serum anti-PLA2R+ Partial remission, 29.5%; complete remission, 25.6% Serum anti-PLA2R−: Partial remission, 23%; complete remission, 42.6% SAb−/GAg− Partial remission, 21.4%; complete remission, 35.7% SAb−/GAg+ Partial remission, 45.4%; complete remission, 27.3% SAb <150/GAg− Partial remission, 42.1%; complete remission, 26.3% SAb <150/GAg+ Partial remission, 19.4%; complete remission, 29% SAb ≥150/GAg− Partial remission, 50%; complete remission, 50% SAb ≥150/GAg+ Partial remission, 33.3%; complete remission, 25%
Zhang (2019)^45^	China	Retrospective cohort study	89 (single)	Epidemiology: Proportion of anti-PLA2R+ patients: 80%
				Clinical Serum anti-PLA2R+ Partial remission, 50%; complete remission, 37.5% Serum anti-PLA2R− Partial remission, 33.3%; complete remission, 66.7% Serum anti-PLA2R+ CTX Partial remission, 59.1%; complete remission, 27.3% Serum anti-PLA2R+ TAC Partial remission, 30%; complete remission, 60% Serum anti-PLA2R− CTX Partial remission, 40%; complete remission, 60% Serum anti-PLA2R− TAC Partial remission, 0%; complete remission, 100%

Abbreviations: Ab, antibody; AKI, acute kidney injury; CTX, cyclophosphamide; DSP, Disease Specific Programme; ED, emergency department; ER, emergency room; ESRD, end-stage renal disease; GAg, glomerular PLA2R antigen; HADS-A, Hospital Anxiety and Depression Scale-Anxiety; HADS-D, Hospital Anxiety and Depression Scale-Depression; HCC, high-cost cohort; HCRU, health care resource utilization; HRQoL, health-related quality of life; IQR, interquartile range; LN, lupus nephritis; LoS, length of stay; MAX, Medicaid Analytic eXtract billing claims; MCO, managed care organization; MN, membranous nephropathy; NA, not applicable; NR, not reported; NHCC, non–high-cost cohort; PLA2R, phospholipase A2 receptor; SAb, serum anti-PLA2R antibody; SD, standard deviation; SLE, systemic lupus erythematosus; TAC, tacrolimus; UK, United Kingdom; US, United States; USRDS, US Renal Data System.

^a^Estimated, patients with SLE, n = 11 663, and patients with SLE and LN, n = 2916.

^b^Number of infections. Described as risk factors for time to first infection-related acute care event (hospitalization or emergency department visit).

^c^HCRU data reported as the average number of visits.

^d^Anti-PLA2R analysis was not available during the earlier years of the study period, and consequently, anti-PLA2R results were only available for 57 patients. Of those tested, 59.6% were positive.

### Epidemiology

**Lupus nephritis:** In total, 5 relevant studies reporting on the epidemiology of LN were identified (**Table 1, Supplementary Table S3**).^13–17^ All were US retrospective cohort studies using Optum (n = 2), LUMEN (n = 1), and Medicaid (n = 2, different counties); data spanned 1976-2019.

Published incidence and prevalence data were identified from US studies only (**Table 1**). Estimates vary between studies, but an average US incidence of 1.3:100 000 was reported between 1976 and 2018.^13^ Incidence nearly doubled over time (0.7 per 100 000 in 1976-1989 to 1.3 in 2000-2018) and prevalence rose from 16.8:100 000 (1985) to 21.2:100 000 (2015), but LN remains rare.^13^ The incidence of LN appeared to be higher in people of older age, females, and African Americans (**Table 1**).^13,17^

The proportion of patients with SLE presenting with LN ranged from 20%^16^ to 38%,^15^ suggesting that LN is among the most prevalent organ-specific manifestations of SLE.^13–16^

**Membranous nephropathy:** Nine relevant studies reporting on the epidemiology in patients with MN were identified, of which 2 were DARs (**Table 1**, **Supplementary Table S7**).^26,37–39,41–45^ Five studies reported data from China, 2 reported US data, 2 reported UK data, and 1 reported data from France (1 study reported data for both the US and France).

MN is a rare disease. A DAR reported the annual incidence of MN to be 10 to 12 per million in North America and 2 to 17 per million in Europe.^39^ However, a study in central China found that the prevalence of MN in patients with biopsy-proven renal diseases increased significantly and nearly doubled from 16% in 2009-2013 to 30.8% in 2014-2018.^37^ A UK study found MN is not a leading diagnosis among renal transplant recipients (3.9%).^26^

Most patients with MN are adults. The only study that included epidemiology for pediatric patients with MN noted that most patients (96.4%) were adults (aged >19 years).^37^ None of the identified studies reported whether geographic variation in local diagnostic or biopsy practices influenced MN diagnosis and, consequently, reported incidence rates.

**Biomarkers:** Four retrospective cohort studies (1 UK, 3 China) reported results by phospholipase A2 receptor (PLA2R) status; PLA2R antibody (PLA2RAb)-positive proportions ranged from 59.6% to 80%.^41,42,44,45^ A DAR reported that antibodies against PLA2R are found in approximately 70% of adult patients with MN and are increasingly being considered a prognostic biomarker.^39^ Other identified biomarkers include thrombospondin type 1 domain-containing 7A (THSD7A), which accounts for less than 5% of primary MN,^39^ although none of the retrospective studies reported THSD7A biomarker epidemiology results.

**Gaps identified from current literature:** LN epidemiological data were identified only for the US, with no data for other countries in scope, regional variation, specific populations, or biomarkers. Evidence was more limited for MN, identified from 4 countries (China, the US, the UK, and France), with reasons for between-study variation unclear; only 1 study reported pediatric data.

### Clinical Burden

**Lupus nephritis:** In total, 19 relevant studies were identified reporting on clinical burden (ie, ESRD, survival/mortality, and healthcare utilization [clinical aspects]) in patients with LN (**Table 1, Supplementary Table S4**).^13–15,18–33^ All were retrospective cohort studies using multicenter or single-center registry data from the US (n = 12), the UK (n = 4), and China (n = 3); data spanned 1975-2019, with sample sizes from 40 to 21 251.

*Progression to ESRD:* A considerable proportion of patients with LN progress to ESRD; however, there was a large degree of variation in the data observed across studies. Eleven relevant studies reported on the proportion of patients with LN progressing to ESRD at different follow-up time points: <5 years (n = 4); 5-10 years (n = 5); and ≥10 years (n = 2). The proportion progressing within 5 years ranged from 5.1% to 23.1%. Population differences contribute, as the study reporting 23.1% included 78% African American patients whereas that reporting 5.1% was a single Chinese center. Other sources of heterogeneity could not be disentangled: data spanned 1975 to 2019, across which regimens changed, baseline severity and proportions with proliferative classes differed, histological classification and ESRD definitions were not uniform, and follow-up varied. Single-center cohorts may reflect referral patterns rather than population risk, so the range should not be read as a single estimate.^15,18,31,32^ Of the 2 studies reporting the proportion of patients who progressed after ≥10 years, 1 reported 25% at 12 years,^22^ and the other reported 33.5% at 42 years.^24^ Patients with Class III and IV LN were more likely to progress to ESRD (25% of patients) than those with Class III + V or IV + V (14%).^22^

*Survival*: Survival findings were consistent across studies. Eight studies reported survival at different time points, the longest at 20 years.^13,20,22,23,26,28,29,31^ Overall, the 5-year survival rates of patients with LN ranged from 89% to 94%^13,22^ and at 10-year follow-up from 70% to 88%.^13,26,29^

Four studies reported on survival across different subgroups of patients with LN. Clinical factors associated with lower survival rates were early (defined as onset before the age of 50 years) vs late-onset LN (10-year survival rates of 50.5% [early onset] vs 84.6% [late onset]^20^), hypertension (66.5% at 15 years follow-up vs 81.3% for patients with normal blood pressure^31^), and acute kidney injury (AKI; AKI-1: 81.8%, AKI-2: 44.9%, AKI-3: 14.6% vs 94.5% for those with no AKI at 10 years’ follow-up^23^). Another study found a lower survival rate for African Americans than Asians at a 10-year follow-up (49% vs 67%, respectively).^28^

*Healthcare resource use*: Healthcare resource use (HCRU) is substantial in LN. Five US studies reported HCRU (inpatient stays, outpatient visits, emergency room [ER] visits, pharmacy claims).^14,25,27,30,33^ Healthcare cost data are presented in the section on economic burden. Patients with LN required more ER visits (47.5% vs 18.5%) and inpatient stays (48% vs 9%) than demographically and clinically matched healthy individuals.^30^

Two studies compared patients with SLE with LN vs without LN and reported that patients with SLE and LN required more inpatient stays (~40% vs ~22%) and ER visits (~60% vs 43%) than those without LN.^14,33^ Another study found females with LN were more likely to require ER visits (952 vs 60 events), outpatient visits (1829 vs 134 events), and hospitalizations (254 vs 44 events) than males with LN.^25^ Among hospitalized patients with LN, 57.8% had 1 infection, 21.1% had 2, and 21.1% had 3 or more.^27^

**Membranous nephropathy**: Seven relevant studies were identified reporting on clinical burden in patients with MN; most studies reported data on ESRD and/or graft failure rates (**Table 1**, **Supplementary Table S8**).^26,40–45^ Four retrospective cohort studies reported results by PLA2R biomarker status (1 study from the UK and 3 from China).^41,42,44,45^

*Remission and relapse*: Complete and partial responses were the most used endpoints across studies that reported clinical outcomes (**Table 1**, **Supplementary Table S8**). However, the definition of complete and partial response differed across studies (**Supplementary Table S10**). Only 1 study included long-term outcomes and had a follow-up of 59.5 months.^41^ That study reported that 33.7% of patients with MN achieved spontaneous (partial or complete) remission, and 41.6% achieved remission after immunosuppression. Of all patients who achieved remission, 42.5% relapsed.^41^ Patients who were PLA2R negative experienced a higher complete remission rate than patients who were PLA2R positive (**Supplementary Figure S2**, **Supplementary Table S8**).

*Progression to ESRD, graft failure, and mortality*: A US study found that approximately 15.9% of patients with primary MN progressed to ESRD.^40^ A small proportion of patients with MN received a kidney transplant (4%),^40^ and the UK posttransplant graft failure and mortality rates have been noted to be high (20% and 16%, respectively).^26^ ESRD, dialysis, and graft outcomes were not studied by PLA2RAb status in the included studies.

*Treatment patterns:* Four studies reported proportions of patients who received immunosuppressant therapy (**Supplementary Figure S3a**).^40–42,45^ The proportion of patients receiving immunosuppressant therapy ranged from 42.7% to 80.7% and is likely to depend on characteristics of patients studied; some studies may include patients who are more likely to achieve spontaneous remission and who do not require treatment.^40–42,45^ Larger proportions of PLA2RAb-positive patients received immunosuppressive treatment compared with the overall population. Treatment patterns in studies reported from China differed from those reported in the US (**Supplementary Figure S3b-S3d**). No treatment pattern by line of therapy was identified.

**Gaps identified from current literature:** Similar to epidemiology, clinical data were limited and geographically restricted, with substantially more evidence for LN than MN. Few studies across either condition reported follow-up sufficient to characterize progression to ESRD, markers of progression, or prognostic factors. For MN, evidence was especially limited for clinically relevant subgroups such as PLA2R-positive and previously transplanted patients, despite indications of higher burden.

### Humanistic Burden

**Lupus nephritis:** Three relevant studies (2 cross-sectional and 1 case-control study) reporting on the humanistic burden (ie, health-related QoL [HRQoL], emotional impact, and treatment satisfaction) in patients with LN were identified (**Table 1**, **Supplementary Table S5**).^34–36^ Included studies were based entirely in the US (n = 1), entirely in China (n = 1), or at multiple global sites (n = 1).

*HRQoL:* One study reported HRQoL using the disease-specific LupusPRO.^35^ Patients with SLE and LN were reported to have poor HRQoL, and patients with SLE and active LN have poorer HRQoL than individuals with nonactive LN.^35^ Effects were seen in domains absent from generic or SLE tools (lupus symptoms, medications, procreation, body image), suggesting a disease-specific tool is needed for renal symptoms. Key concerns relating to lupus medications were “bothersome side effects of medications” and “number of medications.”^35^

*Emotional impact of LN:* A Chinese case-control study found that anxiety (according to Hospital Anxiety and Depression Scale-Anxiety [HADS-A]) and depression (HADS-depression) were higher in patients with LN than in those with SLE but without LN (**Table 1**).^34^

*Treatment dissatisfaction:* Mozaffarian et al (2016) reported US survey data on disease control satisfaction. Patients with SLE and LN had poorer HRQoL than those without LN; dissatisfaction was reported by 25% of patients and 33% of nephrologists, associated with LN severity and disease signs and symptoms.^36^

**Membranous nephropathy:** Only one article (a DAR) reporting the humanistic burden of MN was identified.^39^ It noted impaired QoL and functioning vs age- and gender-matched general population individuals, infrequent reporting of patient-reported outcomes in MN trials, and a need for routine HRQoL measurement.

The review also noted 2 studies, 1 by the Cure Glomerulonephropathy Network (CureGN) involving 478 children and 1115 adults with glomerular diseases, including MN,^46^ and another study conducted in patients with primary glomerular disease that reports some HRQoL data.^47^ These studies were not identified in the original searches for the current review (as they did not fit the inclusion/exclusion criteria) but are included as reported by Ronco et al.^39^ In CureGN (28 pediatric, 305 adult MN patients), patient-reported edema, female sex, obesity, and eGFR correlated with HRQoL decrements, and HRQoL was similar across primary glomerular diseases.^46^ Adults reported worse scores than children across domains. The second study found HRQoL lower than in healthy controls and inversely associated with proteinuria.^47^

**Gaps identified from current literature**: Patient-reported outcomes are poorly studied in LN and MN, and no in-depth or longitudinal studies of patient experience were found. For MN, no primary humanistic burden research met the inclusion criteria; the only humanistic evidence was reported secondarily within a DAR and is therefore indirect. Instruments were predominantly generic or SLE-specific rather than renal-specific. Generic measures permit comparison with population norms but may lack sensitivity to renal symptoms (eg, edema, proteinuria-related fatigue, and treatment burden). For LN, only 1 study used a disease-specific instrument (LupusPRO).^35^ However, it was developed and validated for SLE rather than renal involvement, and no identified instrument has documented validity, reliability, or responsiveness in MN, limiting interpretation of reported HRQoL findings. No study related worsening clinical burden to patient-reported outcomes.

### Economic Burden

**Lupus nephritis:** Four relevant economic burden studies (ie, healthcare costs) were identified, all of which were US retrospective cohort studies illustrating a high economic burden (**Table 1**, **Supplementary Table S6**).^14,15,30,33^ Economic burden comparisons were made between patients with SLE with/without LN, patients with LN and matched controls, and patients with different levels of LN disease activity.

Sources were Optum (n = 3) and a managed care organization database (n = 1); data spanned 2003-2019, with sample sizes of 907 to 21 251.

Bell et al (2023) reported a US mean total all-cause annual cost of $50 975 per patient with SLE and LN.^33^ These were significantly higher than for SLE without LN ($26 262; *P* < .001). Bell et al (2022) reported severe flares incurred the highest mean 12-month flare-related costs, at $29 148 per flare.^14^

**Figure 1.**
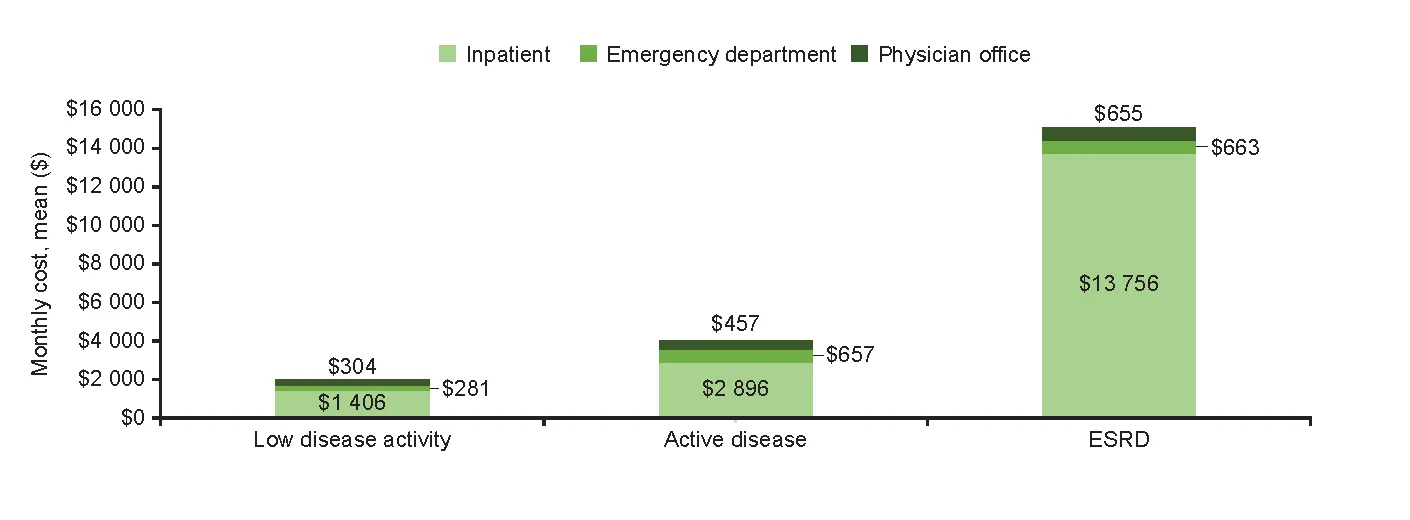
US Health Care Costs Associated with Lupus Nephritis, According to Disease Activity^15^ Abbreviation: ESRD, end-stage renal disease. Data are presented in US dollars.

Another study found US healthcare costs increased with increasing disease severity, from approximately $2000 per month in patients with low disease activity to approximately $15 000 per month for patients with ESRD (**Table 1**, **Figure 1**).^15^ At all levels of disease activity, the greatest costs were incurred due to inpatient stays.^15^ Costs across inpatient, emergency, physician office, and outpatient settings were higher with active disease or ESRD, the greatest difference being inpatient costs. Furst et al reported US costs collected in 2003-2008 and are therefore not presented here.^30^

**Membranous nephropathy:** Only 1 study, a US retrospective observational analysis, studied commercially insured patients with MN (n = 2689) and reported resource use and associated costs (**Table 1**, **Supplementary Table S9**).^40^ Data came from IBM MarketScan (employer-sponsored commercial and Medicare Advantage plans, 2012-2015). Resource use was higher for the high-cost cohort (HCC) than the non-high-cost cohort (NHCC) across all settings over 1 year (4.7 more outpatient visits; 1.7 more inpatient admissions).^40^ According to the average number of claims, outpatient hospital (34.7 vs 7.1 claims) and ESRD facility use (33.3 vs 3.6) were the primary drivers of this difference.^40^ Mean per-patient all-cause healthcare costs for patients with MN were $401 608 for the HCC and $27 154 for the NHCC.^40^

The primary driver of total costs for both cohorts occurred in the outpatient setting (46.6% for HCC and 52.8% for NHCC); costs from outpatient hospital and ESRD facilities comprised 47.4% and 36% of total outpatient costs for HCC and 49.9% and 18.3% for NHCC, respectively.^40^

**Gaps identified from current literature:** Again, data were restricted to a small number of US retrospective cohort studies, with fewer for MN than LN; no cost data were identified for other countries/regions in scope, and no long-term data were found. Estimates were not directly comparable: costs were collected between 2003 and 2019 and reported in each source price year without inflation adjustment or currency standardization, and studies differed in cost categories (all-cause vs disease-attributable; pharmacy, outpatient, and dialysis costs) and health system. Figures are therefore presented as reported rather than as comparable estimates.

## DISCUSSION

This TLR highlights the considerable clinical, humanistic, and economic burden of LN and MN. For both diseases, burden is largely driven by progression to ESRD, which likely underpins costs and impacts on patient QoL, although evidence remains limited. The full burden of disease is poorly characterized, with available evidence derived from heterogeneous populations, restricted geographies, and short follow-up periods.

Epidemiological data for LN were limited to US retrospective studies, and further data are needed to examine global patterns and variations. Available US data suggest that LN affects 20% to 38% of patients with SLE,^15,16^ and that the incidence of LN may increase with age and be higher in women and Black or African American patients.^13,17^ Differences in reported incidence may partly reflect underlying population heterogeneity, as renal involvement in SLE has been shown to occur more frequently among patients of African and Hispanic origin compared with those of White ancestry.^48^ Variation in national ethnic compositions may, therefore, contribute to the wide ranges reported across datasets. However, further studies are needed to confirm this and examine the reasons for the large variations in the datasets identified. MN data were limited but spanned 4 countries (China, the US, the UK, and France).^37–39^ MN remains a rare disease, although reported incidence rates vary for Europe and North America,^39^ and data indicate an increase in both incidence and prevalence in recent years.^37^ No study examined whether geographic variation in MN epidemiology reflects local practice, such as biopsy thresholds or access to diagnostics; this is relevant because included studies used biopsy-proven populations. Up to 80% of patients with MN are reported PLA2RAb positive.^39,41,42,44,45^ Although PLA2RAb positivity is associated with poorer prognosis, its association with clinical remission remains uncertain, with conflicting findings reported across meta-analysis publications.^49^ PLA2RAb testing was not routine practice until its inclusion in the Kidney Disease: Improving Global Outcomes (KDIGO) guidelines in 2021.^11^ Testing is often limited to diagnosis, restricting its use at later stages.

Both LN and MN are associated with high clinical burden and HCRU, with ESRD and mortality being important factors. Across studies, 21.1% to 36.8% of patients with LN progressed to ESRD within 5 to 10 years^13,19,21,25,29^ and 10-year survival ranged from 70% to 88%.^13,26,29^ AKI presence, age at onset, and ethnicity were identified among factors that may impact survival and should be considered in LN trial eligibility criteria or subgroup analyses.^20,23,28,31^ Substantially fewer studies evaluated clinical burden in MN, which may reflect its more favorable prognosis and less intensive follow-up but may also mean burden is underrecognized in those who do progress, particularly PLA2R-positive patients. MN prognosis is variable, with PLA2R-negative patients more likely to achieve complete remission.^41^ Inconsistent remission definitions limit comparability across studies. PLA2R-positive patients are also more likely to receive immunosuppressive treatment compared with the overall population.^41^ However, no clear first-line treatment pattern was identified, and treatment patterns differed between China and the US.^40–42,45^

Progression to ESRD was reported in only 1 US study of MN, where approximately 15.9% patients with primary MN progressed to ESRD.^40^ Although a small proportion of patients received a kidney transplant (4%),^40^ a UK study reported high graft failure and mortality rates for posttransplant patients (20% and 16%, respectively).^26^ Longer-term follow-up is needed to examine the true burden of ESRD by PLA2R status and the relationship between clinical response as an intermediate marker for ESRD.

Humanistic burden data are limited, but available reports suggest poorer HRQoL in patients with LN or MN vs the general population. Renal-specific instruments and routine HRQoL assessment would be required to characterize humanistic burden, and may capture concerns such as medication burden in LN.^35^

Economic burden evidence was limited to US studies, with high costs reflecting the high clinical burden of LN and MN. Costs are driven by ESRD, which is associated with monthly costs of approximately $15 000 per patient with LN in the US.^15^ Active disease and flares were also drivers of costs for LN,^15,33^ although inpatient stays incurred most costs at all levels of disease.^15^ Only 1 study reported costs for MN, where most costs occurred in the outpatient setting, particularly for outpatient hospital and ESRD facilities.^40^ The scarcity of economic data for MN mirrors clinical evidence gaps and may further underestimate long-term costs in patients who experience progression. Given that costs are concentrated among patients with ESRD and active disease, the evidence is consistent with a relationship between progression and expenditure, although the included studies were not designed to evaluate any intervention effect on costs.

### Impact of Evidence Gaps and Priorities for Future Research

Limited data across countries constrained assessment of burden between regions and subpopulations. Gaps were most pronounced for MN, where few studies were identified and data were often restricted to subgroups more likely to progress, such as PLA2R-positive or transplanted patients; overall MN burden may therefore be underestimated.

Gaps in the identified literature limit understanding of disease progression, patient experience, and economic impact. Reasons for the evidence gaps require further consideration. The evidence base for LN and MN is fragmented, and the absence of accessible local and national-level databases and registries constrains the ability to capture real-world treatment patterns, long-term outcomes, and regional variation in clinical practice. Differences in ethnic composition across national populations may also contribute to epidemiological variation, given the higher renal dysfunction rates shown among certain ethnic groups.^48^

The lack of natural history and real-world evidence studies limits understanding of long-term disease trajectories, treatment sequencing, and routine clinical decision-making. This may contribute to a disconnect between clinical guidelines and real-world care, as recommendations rest largely on trial data rather than long-term observational evidence.

Previous HTA submissions have criticized the lack of long-term efficacy data, including ESRD (number of dialysis sessions and/or transplants).^12^ As these outcomes typically emerge 5 to 10 years after diagnosis, longer-term studies and international disease registries capable of following patients beyond the time horizons captured in existing studies are required to generate more robust epidemiological evidence.

Longitudinal data covering diagnosis through ESRD would support evaluation of symptoms, QoL, and HCRU over time and help validate markers of progression, for both high-risk MN subgroups and the broader population.

Research priorities, therefore, include longitudinal follow-up across geographies and understanding the patient journey from diagnosis to ESRD; studies stratified by ethnicity to support cross-country comparison; markers of rapid progression; validated renal-specific patient-reported outcome instruments; standardized definitions of remission, relapse, and ESRD; and cost analyses using consistent price years and categories.

### Limitations

This review was targeted rather than systematic, and the search was restricted to MEDLINE and EMBASE, English-language publications, 6 predefined countries, and records from January 2013 to August 2023, with no registered protocol. Scopus, the Cochrane Library, and regional databases were not searched. A broader multi-database search would likely identify further records, so the evidence described is that retrieved under these restrictions rather than the complete evidence base.

The absence of formal risk-of-bias or quality appraisal is a major limitation of this review. Included evidence was predominantly retrospective cohort studies, registry analyses, cross-sectional studies, and surveys, designs susceptible to selection and confounding bias, and several relied on administrative claims where ascertainment depends on coding practice. Without appraisal it cannot be determined whether findings are robust or influenced by lower-quality studies, and no study was weighted on quality grounds. Reported ranges should be read as the span of published estimates rather than an appraised summary of risk.

The search ran in August 2023 with no supplementary search thereafter. Evidence published since, including new epidemiological, outcome, and cost studies and subsequent regulatory or reimbursement decisions, is not captured, and estimates may have been updated. The findings remain informative for the objective of identifying structural gaps rather than current point estimates: the gaps identified are longstanding features of this literature, including concentration of epidemiological and economic data in the US, scarcity of primary humanistic evidence in MN, absence of renal-specific patient-reported outcome instruments, lack of standardized definitions of remission, relapse, and ESRD, and limited follow-up beyond 5-10 years. Quantitative findings should nonetheless be treated as a snapshot of literature available to August 2023.

The 6 countries were selected pragmatically rather than by burden, and countries with substantial burden, including lower- and middle-income settings, were not examined, so descriptions of burden should not be read as global in coverage. No eligible studies were retrieved for Germany or Japan, reflecting absence of studies meeting the criteria under the applied restrictions rather than absence of disease burden or national literature. Restriction to English-language publications is a limitation because China and Japan were within scope and relevant nephrology literature from both is published in Chinese and Japanese; evidence from these settings is likely underrepresented.

Interventional studies were excluded because the review characterized real-world burden. This is a limitation, as trial publications may report quality of life and other patient-reported outcomes not captured here, and humanistic evidence was sparse for LN and absent as primary research for MN. Relevant evidence may therefore exist in the interventional literature outside this scope.

The evidence identified for MN was small in volume, particularly for clinical, humanistic, and economic burden, where several observations rest on single studies or data reported secondarily within DARs. MN findings should be regarded as indicative rather than established, and the apparent LN-MN contrast may partly reflect differences in available evidence rather than underlying burden. Economic findings are further limited by absent inflation adjustment and heterogeneity in price years, cost categories, payer perspectives, and health systems; no quantitative synthesis was attempted.

## CONCLUSIONS

While available data show the significant clinical, economic, and humanistic burden of LN and MN, especially for patients with ESRD, major evidence gaps remain. Longitudinal data covering the full disease course would help address these gaps. Better characterization of disease burden would clarify long-term outcomes, patient experience, and economic impact, supporting evidence-based decision-making. As this is a targeted review of literature published to August 2023, restricted by language, geography, and study design, and conducted without formal quality appraisal, these findings describe the evidence identified rather than all evidence available. Further country-specific data would support assessment of burden across populations and health systems.

### Disclosures

F.B. is an employee of argenx. V.P., A.R., and C.K. are affiliated with Avalere Health, which received funding from argenx for this work.

## Supporting information

Online Supplementary Material
