# Supplementary material for: Understanding the Evidence Gaps in the Epidemiology and Burden of Lupus Nephritis and Membranous Nephropathy: A Targeted Literature Review
Source: J Health Econ Outcomes Res. 2026 Oct 7;13(2):120–34. doi: 10.36469/001c.166255 (PMC13651649; doi:10.36469/001c.166255)
Supplement: Supplementary file 1 [file jheor_2026_13_2_166255_370173.pdf]

Journal of Health Economics and Outcomes Research

Online Supplementary Material

Understanding the Evidence Gaps in the Epidemiology and Burden of Lupus Nephritis and Membranous Nephropathy: A Targeted Literature Review. *JHEOR*. 2026;13(2):120-134. doi:10.36469/jheor.2026.166255

Figure S1: PRISMA Diagrams **2**

Figure S2: Complete Remission Rates for Patients with Membranous Nephropathy According to PLA2R Status **3**

Figure S3: Immunosuppressant Therapy in Patients with Membranous Nephropathy **4**

Table S1: Study Eligibility Criteria **5**

Table S2: Search Strategy **9**

Table S3: Characteristics of Studies Reporting on Lupus Nephritis Epidemiology **12**

Table S4: Characteristics of Studies Reporting on the Clinical Burden of Lupus Nephritis **13**

Table S5: Characteristics of Studies Reporting on the Humanistic Burden of Lupus Nephritis **17**

Table S6: Characteristics of Studies Reporting on the Economic Burden of Lupus Nephritis **19**

Table S7: Characteristics of Studies Reporting on Membranous Nephropathy Epidemiology **21**

Table S8: Characteristics of Studies Reporting on the Clinical Burden of Membranous Nephropathy **23**

Table S9: Characteristics of Studies Reporting on the Economic Burden of Membranous Nephropathy **27**

Table S10: Variations in Definitions of Complete and Partial Response Used for Patients with Membranous Nephropathy **28**

This supplementary material has been provided by the authors to give readers additional information about their work.

This is an open-access article distributed under the terms of the Creative Commons Attribution 4.0 International License (CCBY-4.0). View this license's legal deed at http://creativecommons.org/licenses/by/4.0 and legal code at http://creativecommons.org/licenses/by/4.0/legalcode for more information.

Figure S1. PRISMA Diagrams

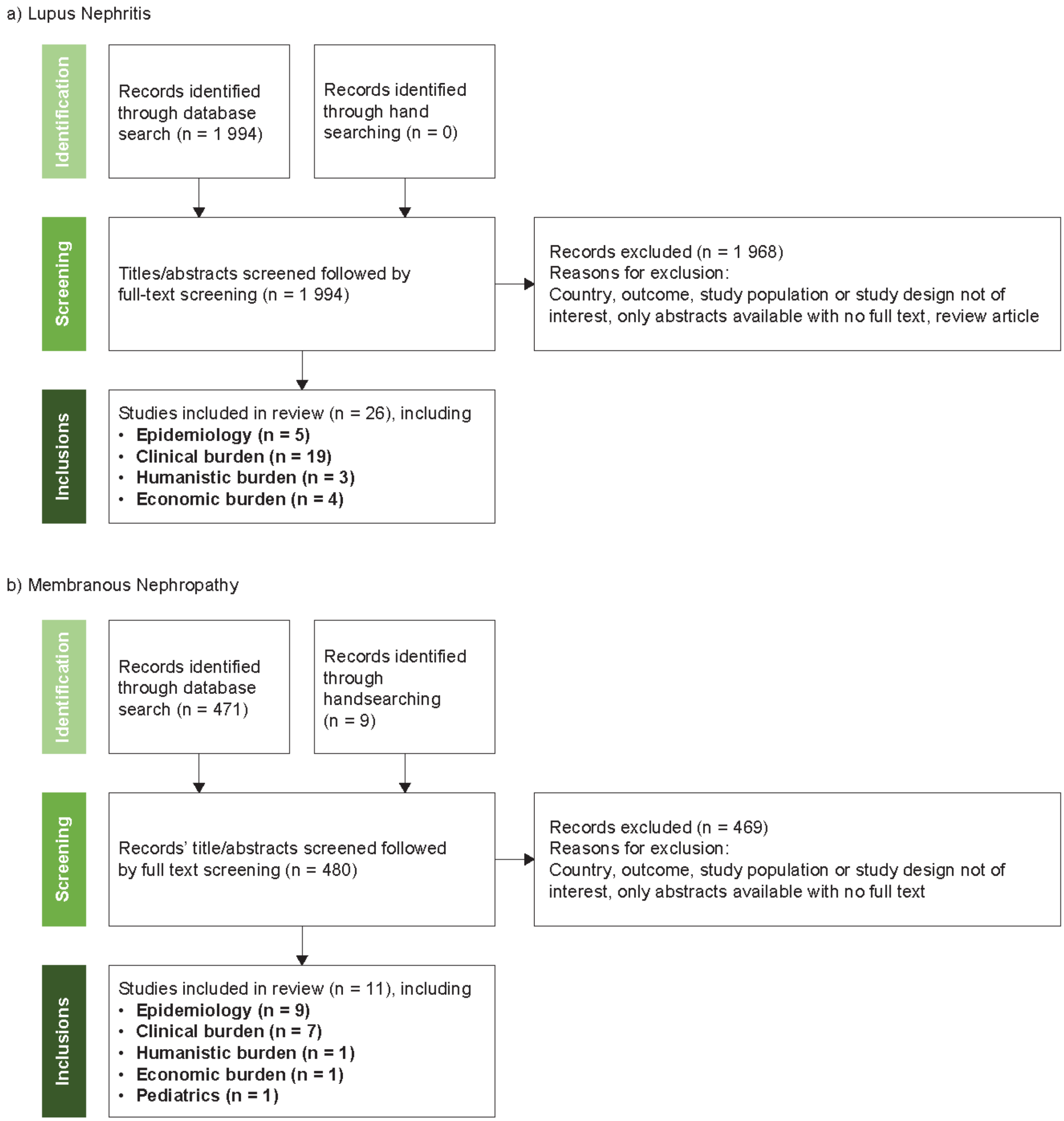

Note: Some studies included data for more than one research question.

Figure S2. Complete Remission Rates for Patients with Membranous Nephropathy According to PLA2R Status

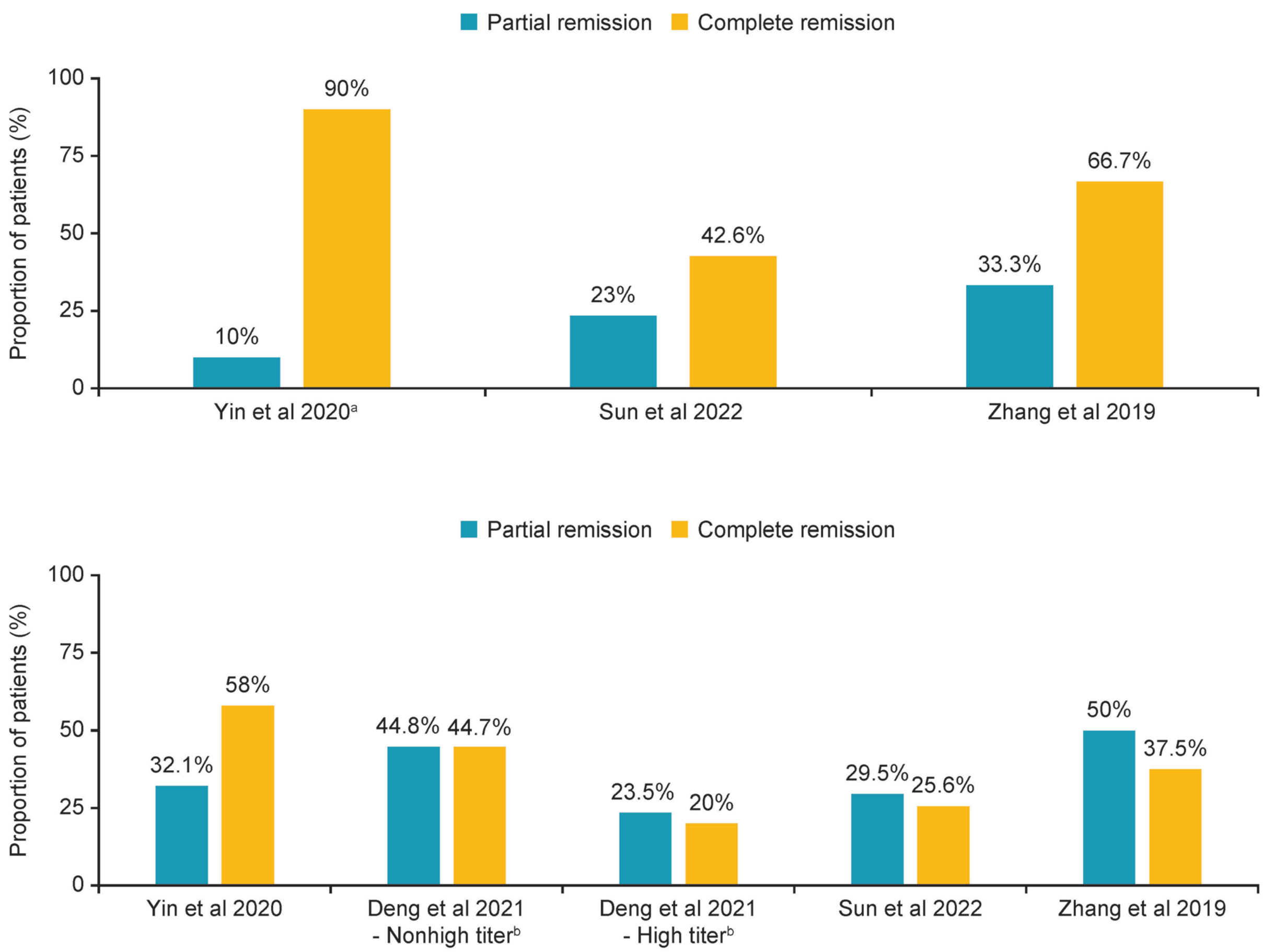

Abbreviation: PLA2R, phospholipase A2 receptor.
Upper panel: Analysis of proportion of PLA2R-negative patients with complete and partial remission at 12-month follow-up.
Lower panel: Analysis of proportion of PLA2R+ patients with complete and partial remission at 12-month follow-up.
[a]Data from Yin et al (2020) in the PLA2R-negative group is reported from the group of patients that were negative for serum antibody and negative for glomerular antigen.
[b]Deng et al (2021) only reported outcomes from PLA2R+ patients. It defined high titer as patients with PLA2R level >150 RU/mL and non-high titer as PLA2R level between 20 and 150 RU/mL.

Figure S3. Immunosuppressant Therapy in Patients with Membranous Nephropathy

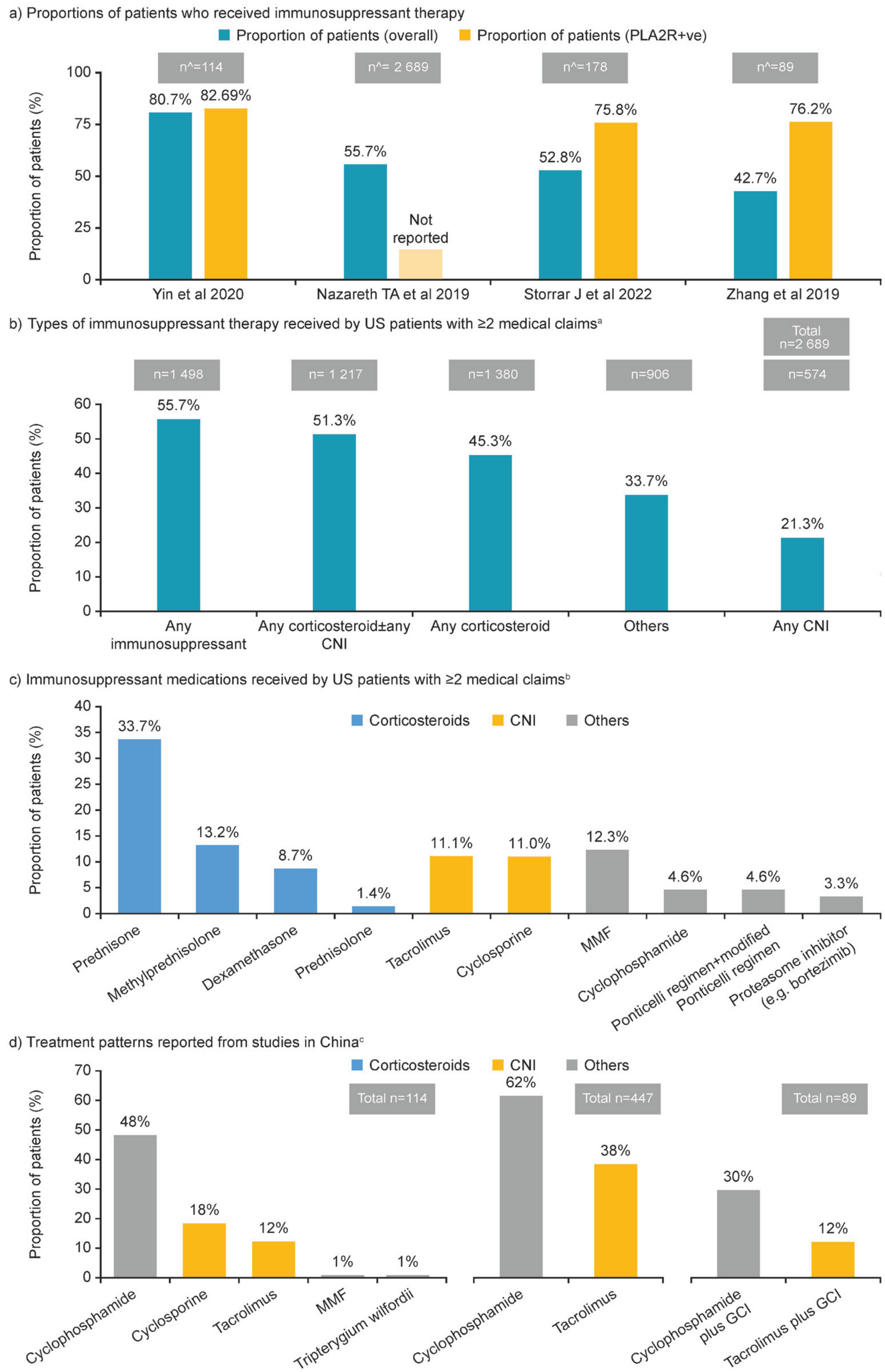

Abbreviations: CNI, calcineurin inhibitor; GCI, glucocorticoid; IVIG, intravenous immunoglobulin; MMF, mycophenolate mofetil; PLA2R, phospholipase A2 receptor; RCI, repository corticotropin injection.

[a]Treatments shown are not mutually exclusive and, therefore, may not add up to 100%. Others included RCI, N = 62 (2.3%); rituximab, N = 33 (1.2%); IVIG, N = 14 (0.5%); plasmapheresis, N = 6 (0.2%); chlorambucil, N = 4 (0.1%); Ponticelli regimen, N = 4 (0.1%); modified Ponticelli regimen, N = 120 (4.5%); Ponticelli regimen + modified Ponticelli regimen, N = 124 (4.6%); MMF, N = 332 (12.3%); cyclophosphamide, N = 124 (4.6%); and proteasome inhibitor, N = 88 (3.3%).

[b]Other treatments not shown include RCI, N = 62 (2.3%); rituximab, N = 33 (1.2%); IVIG, N = 14 (0.5%); plasmapheresis, N = 6 (0.2%); and chlorambucil N = 4, (0.1%).

[c]Yin et al (2020), Deng et al (2022), Zhang et al (2019), and Nazareth et al (2019) are not comparable studies as they look at different populations and have not been adjusted for baseline characteristics.

Table S1 Study Eligibility Criteria

Lupus nephritis							
**Inclusion criteria**							
**Research question**	**Participants**	**Comparison**	**Outcomes**	**Study design**	**Setting**	**Language**	**Search dates**
**Epidemiology**	Adult patients (aged ≥18 years) with LN	Patients with LN vs without; patients with different types or stages of LN; no comparison	Prevalence, incidence	• Observational studies (prospective, retrospective, cohort, cross-sectional, longitudinal, registry)<br>• SLRs of observational studies<br>• DARs or economic studies to cross-check the completeness of the search	France, Germany, the UK, Japan, China, the US	English	From January 1, 2013 to August 23, 2023
**Clinical burden**			Mortality, morbidity, hospitalization, ESRD, graft loss, dialysis				
**Economic burden**			Direct costs, non–hospital-related costs (eg, pharmacy costs), indirect costs (productivity loss, absenteeism)				
**Humanistic burden**			Physical symptoms (fatigue, pain/discomfort, reduced physical functioning), emotional impact (anxiety/depression, stress, emotional well-being), quality of life	• Observational studies (prospective, retrospective, cohort, cross-sectional, longitudinal, registry)			

				• PRO studies (surveys, interviews, questionnaires), case-control<br>• SLRs of observational studies to cross-check the completeness of the search			
**Exclusion criteria**							
**Research question**	**Participants**	**Comparison**	**Outcomes**	**Study design**	**Setting**	**Language**	**Search dates**
**All (epidemiology and burden)**	Individuals aged <18 years	NA	NA	• Case reports<br>• Case studies	Studies in other countries	Other languages	Studies published before January 1, 2013
**Membranous nephropathy**							
**Inclusion criteria**							
**Research question**	**Participants**	**Comparison**	**Outcomes**	**Study design**	**Setting**	**Language**	**Search dates**
**Epidemiology**	• Adult patients (aged ≥18 years) with primary MN	• Patients with pMN vs without; patients with	Prevalence, incidence	• Observational studies (prospective, retrospective, cohort, cross-sectional, longitudinal, registry)	France, Germany, the UK, Japan, China, the US	English	From January 1, 2013 to August 23, 2023
**Clinical burden**			Mortality, morbidity, hospitalization, ESRD				
**Economic burden**			Direct costs, non–hospital-related costs (eg, pharmacy costs),				

	• Individuals aged <18 years with primary MN	different types of pMN; no comparison	indirect costs (productivity loss, absenteeism)	• SLRs of observational studies<br>• DARs or economic studies to cross-check the completeness of the search			
**Humanistic burden**			Physical symptoms (fatigue, pain/discomfort, reduced physical functioning), emotional impact (anxiety/depression, stress, emotional well-being), quality of life	• Observational studies (prospective, retrospective, cohort, cross-sectional, longitudinal, registry)<br>• PRO studies (surveys, interviews, questionnaires), case-control<br>• SLRs of observational studies to cross-check the			

				completeness of the search			
**Exclusion criteria**							
**Research question**	**Participants**	**Comparison**	**Outcomes**	**Study design**	**Setting**	**Language**	**Search dates**
**All (epidemiology and burden)**	Individuals with secondary MN	NA	NA	• Case reports<br>• Case studies	Studies in other countries	Other languages	Studies published before January 1, 2013

Abbreviations: DAR, disease area review; ESRD, end-stage renal disease; LN, lupus nephritis; MN, membranous nephropathy; NA, not applicable; pMN, primary membranous nephropathy; PRO, patient-reported outcome; SLR, systematic literature review; UK, United Kingdom; US, United States.

Table S2 Search strategy

EMBASE searches: August 24, 2023		
**Lupus nephritis**		
**No.**	**Query**	**Results**
#1	'lupus nephritis'/exp OR 'lupus nephritis' OR 'systemic lupus erythematosus nephritis' OR 'sle nephritis' OR 'kidney involvement in lupus' OR 'renal complications in lupus'	23 739
#2	'epidemiology'/de OR 'prevalence'/de OR 'incidence'/de OR 'register'/de OR 'disease registry'/de	1 784 519
#3	(epidemiol* NEAR/1 (study OR studies OR trial OR trials OR survey OR surveys OR review)):ti,ab	144 757
#4	((direct OR indirect OR healthcare OR 'health care') NEAR/5 (cost OR costs)) OR ((opportunity OR societal OR wider OR indirect OR employer OR patient OR carer OR caregiver) NEXT/1 (cost OR costs))	294 913
#5	'ambulatory care'/exp OR 'outpatient department'/de OR 'outpatient'/de OR ambulatory:ti,ab,de OR outpatient:ti,ab,de	555 615
#6	'hospital emergency service'/de OR 'emergency department*':ti,ab OR 'emergency service*':ti,ab OR 'emergency room':ti,ab OR 'accident and emergency':ti,ab OR 'accident & emergency':ti,ab	244 909
#7	'hospitalization'/de OR 'hospital stay*' OR hospitali?ation OR 'hospital admission*' OR ((hospital OR gp OR practitioner) NEXT/1 visit*) OR 'hcru' OR 'healthcare resource utilization'	963 563
#8	productivity NEXT/1 (loss OR lost OR losses OR lose)	5 351
#9	((employment OR work OR school) NEAR/5 (absence* OR absent*)) OR absenteeism OR unemployment	61 359
#10	'morbidity'/de OR morbidity OR 'comorbidity'/de OR comorbid* OR 'mortality'/de OR 'mortality' OR 'burden'	3 060 635
#11	'quality of life'/de OR 'quality of life index'/de OR 'quality adjusted life year'/de OR 'outcome assessment'/de OR 'disability'/de OR 'daily life activity'/de OR 'health status'/de OR 'patient satisfaction'/de OR 'severity of illness index'/de OR 'disease severity'/de	2 486 817
#12	#2 OR #3 OR #4 OR #5 OR #6 OR #7 OR #8 OR #9 OR #10 OR #11	7 257 351
#13	#1 AND #12	7 295
#14	'in vitro study'/exp OR 'case report' OR 'case study' OR editorial OR 'in vitro' OR 'editorial'/de OR 'letter'/de OR 'case report'/de	12 298 552
#15	#13 NOT #14	4 279
#16	#13 NOT #14 AND [article]/lim AND [english]/lim AND [2013-2023]/py	1 143
**Membranous nephropathy**		
**No.**	**Query**	**Results**
#1	'membranous nephropathy' OR 'idiopathic membranous nephropathy' OR 'primary membranous nephropathy' OR 'secondary membranous nephropathy' OR 'membranous glomerulopathy'	7 515
#2	'epidemiology'/de OR 'prevalence'/de OR 'incidence'/de OR 'register'/de OR 'disease registry'/de	1 784 519
#3	(epidemiol* NEAR/1 (study OR studies OR trial OR trials OR survey OR surveys OR review)):ti,ab	144 757
#4	((direct OR indirect OR healthcare OR 'health care') NEAR/5 (cost OR costs)) OR ((opportunity OR societal OR wider OR indirect OR employer OR patient OR carer OR caregiver) NEXT/1 (cost OR costs))	294 913
#5	'ambulatory care'/exp OR 'outpatient department'/de OR 'outpatient'/de OR ambulatory:ti,ab,de OR outpatient:ti,ab,de	555 615
#6	'hospital emergency service'/de OR 'emergency department*':ti,ab OR 'emergency service*':ti,ab OR 'emergency room':ti,ab OR 'accident and emergency':ti,ab OR 'accident & emergency':ti,ab	244 909

#7	'hospitalization'/de OR 'hospital stay*' OR hospitali?ation OR 'hospital admission*' OR ((hospital OR gp OR practitioner) NEXT/1 visit*) OR 'hcru' OR 'healthcare resource utilization'	963 563
#8	productivity NEXT/1 (loss OR lost OR losses OR lose)	5 351
#9	((employment OR work OR school) NEAR/5 (absence* OR absent*)) OR absenteeism OR unemployment	61 359
#10	'morbidity'/de OR morbidity OR 'comorbidity'/de OR comorbid* OR 'mortality'/de OR 'mortality' OR 'burden'	3 060 635
#11	'quality of life'/de OR 'quality of life index'/de OR 'quality adjusted life year'/de OR 'outcome assessment'/de OR 'disability'/de OR 'daily life activity'/de OR 'health status'/de OR 'patient satisfaction'/de OR 'severity of illness index'/de OR 'disease severity'/de	2 486 817
#12	#2 OR #3 OR #4 OR #5 OR #6 OR #7 OR #8 OR #9 OR #10 OR #11	7 257 351
#13	#1 AND #12	1 701
#14	'in vitro study'/exp OR 'case report' OR 'case study' OR editorial OR 'in vitro' OR 'editorial'/de OR 'letter'/de OR 'case report'/de	12 298 552
#15	#13 NOT #14	780
#16	#13 NOT #14 AND [article]/lim AND [english]/lim AND [2013-2023]/py	224
**MEDLINE searches: August 30, 2023**		
**Lupus nephritis**		
**No.**	**Query**	**Results**
#1	"lupus nephritis"[MeSH Terms] OR "lupus nephritis" OR "systemic lupus erythematosus nephritis"	12 130
#2	("epidemiology"[MeSH Terms] OR "prevalence"[MeSH Terms] OR "incidence"[MeSH Terms])	644 470
#3	direct costs OR indirect costs	41 678
#4	"ambulatory care" [MeSH Terms]	56 507
#5	("Emergency Service, Hospital"[Mesh Terms])	99 622
#6	("Hospitalization"[Mesh Terms])	293 118
#7	productivity OR absenteeism	3 682 163
#8	"Quality of Life"[Mesh Terms]	271 557
#9	"Morbidity"[Mesh Terms]	650 834
#10	(#2) OR (#3) OR (#4) OR (#5) OR (#6) OR (#7) OR (#8) OR (#9)	4 860 710
#11	(#1) AND (#10)	1 728
#12	(#1) AND (#10)	851 [from 2013 – 2023]
**Membranous nephropathy**		
**No.**	**Query**	**Results**
#1	("membranous nephropathy" OR "idiopathic membranous nephropathy" OR "primary membranous nephropathy" OR "secondary membranous nephropathy" OR "membranous glomerulopathy")	5 016
#2	("epidemiology"[MeSH Terms] OR "prevalence"[MeSH Terms] OR "incidence"[MeSH Terms])	644 470
#3	direct costs OR indirect costs	41 683
#4	"ambulatory care" [MeSH Terms]	56 507
#5	("Emergency Service, Hospital"[Mesh Terms])	99 622

#6	("Hospitalization"[Mesh Terms])	293 119
#7	productivity OR absenteeism	3 682 532
#8	"Quality of Life"[Mesh Terms]	271 557
#9	"Morbidity"[Mesh Terms]	650 834
#10	(#2) OR (#3) OR (#4) OR (#5) OR (#6) OR (#7) OR (#8) OR (#9)	4 861 083
#11	(#1) AND (#10)	482
#12	(#1) AND (#10)	247 [from 2013 – 2023]

Table S3 Characteristics of studies reporting on LN epidemiology

Study description										Results		
**Author**	**Year**	**Type of study**	**Country**	**Number of centers**	**Data collection year**	**Study groups**	**Sample size of study group**	**Gender (% females)**	**Mean (SD) age (y)**	**Prevalence**	**Incidence**	**% of patients with SLE and LN**
Hocaoglu, M.	2023	Retrospective cohort study	USA	LUMEN registry	1976-2018	LN	72	76.4	38.4 (16.2) at diagnosis	In 2015: 21.2 per 100 000	In 2000-2018: 1.3 per 100 000	NR
Bell, C.F.	2022	Retrospective cohort study	USA	Optum	2015-2019	LN	2 916	83.5	65.0 (14.9) at diagnosis	NR	NR	25[a]
Dall'Era, M.	2023	Retrospective cohort study	USA	Optum	2007-2019	LN	21 251	86.9	60.3 (15.7) at baseline	Prevalent cases: 46.5%	Incident cases: 53.6%	37.9
Tedeschi, S.K.	2019	Retrospective cohort study	USA	Medicaid	2007-2010	LN	9 449	NR	NR	NR	NR	19.8
Feldman, C.H.	2013	Retrospective cohort study	USA	Medicaid	2000-2004	LN	7 388	90.0	NR	30.9 per 100 000 (2000-2004)	Average incidence rate: 6.85 per 100 000 person-years (2002-2004)	21.5

[a]Estimated, patients with SLE n = 11 663 and patients with SLE and LN n = 2 916
LN, lupus nephritis; NR, not reported; SD, standard deviation; SLE, systemic lupus erythematosus

Table S4 Characteristics of studies reporting on the clinical burden of LN

Study description										Results		
**Author**	**Year**	**Type of study**	**Country**	**Number of centers**	**Data collection year**	**Study groups**	**Sample size of study group**	**Gender (% females)**	**Mean (SD) age (y)**	**% ESRD**	**Survival/mortality**	**Healthcare utilization**
Khosroshahi, A.	2023	Retrospective cohort study	USA	Five	2010-2020	LN	173	86.1	Median 31 (IQR 24–40)[a] at diagnosis	3-year: 23.1	3-year mortality: 6.1%	NR
Duong, M.D.	2023	Retrospective cohort study	USA	Single	2005-2018	LN	125	84.0	NR	5-year: 45.7	NR	NR
Hocaoglu, M.	2023	Retrospective cohort study	USA	LUMEN	1976-2018	LN	72	76.4	38.4 (16.24) at diagnosis	5-,10-year: 10, 13	5-,10-year survival: 89, 70	NR
Bell, C.F.	2022	Retrospective cohort study	USA	Optum	2015-2019	LN	2 916	83.5	65 (14.9) at diagnosis	NR	NR	Inpatient stays: 1 195 (41%) Outpatient visits: 2 916 (100%) ER visits: 1 686 (57.8%) Pharmacy claims: 2 723 (93.4%)
Dall'Era, M.	2023	Retrospective cohort study	USA	Optum	2007-2019	LN	21 251	86.9	60.3 (15.7) at baseline	10.5 (mean follow-up 30.6 months)	NR	NR
Tian, N.	2022	Retrospective cohort study	China	Single	1996-2011	All LN	1 264	~83	31.1 (12.2) at diagnosis	NR	NR	NR
						Early-onset LN	1 162	~83	28.8 (10) at diagnosis		5-year survival: 67.6 10-year survival: 50.5 $p < 0.001$	
						Late-onset LN	102	~71%	58.1 (6.8) at diagnosis		5-year survival: 89.8 10-year survival: 84.6 $p < 0.001$	

Study description										Results		
**Author**	**Year**	**Type of study**	**Country**	**Number of centers**	**Data collection year**	**Study groups**	**Sample size of study group**	**Gender (% females)**	**Mean (SD) age (y)**	**% ESRD**	**Survival/mortality**	**Healthcare utilization**
Gisca, E.	2021	Retrospective cohort study	UK	Single	1975-2015	LN	219	91.3	27 (16) at diagnosis	5-,10-year: 28.9; 36.8	5-year mortality: 19%	NR
Farinha, F.	2020	Retrospective cohort study	UK	Single	1978-2017	LN Class III + IV	135	91	29 (12) at diagnosis	25	Renal survival, 5, 10, 15, 20-year: 94, 86, 80,76 (cumulative)	NR
						LN Class V	38	87	29 (14) at diagnosis	8		
						Class III/IV+ V	14	79	26 (8) at diagnosis	14 (median follow-up: 12 years)		
Chen, T.	2020	Retrospective cohort study	China	Single	2000-2014	LN + no AKI	82	93	29 (9) at baseline	NR	10-year: 94.5%	NR
						LN + AKI 1	40	98	30 (10) at baseline		10-year: 81.8%	
						LN + AKI 2	26	89	32 (12) at baseline		10-year: 44.9%	
						LN + AKI 3	19	90	30 (12) at baseline		10-year: 14.6%	
Ntatsaki, E.	2018	Retrospective cohort study	UK	Two	1975-2015	LN + transplant	40	85	21.1 (9.2) at diagnosis	42-year: 33.5	5, 8.6-year: 95, 80	NR
Feldman, C.H.	2018	Retrospective cohort study	USA	MAX	2000-2010	LN + female	2 467	100 (90% of total cohort)	29.6 (13.9) at diagnosis	5-year: 21.1	5-year cumulative incidence of death: 9.4 (95% CI 5.4–14.9)	Inpatient stays: 254 Outpatient stays: 1 829 ER visits: 952

Study description										Results		
Author	Year	Type of study	Country	Number of centers	Data collection year	Study groups	Sample size of study group	Gender (% females)	Mean (SD) age (y)	% ESRD	Survival/mortality	Healthcare utilization
						LN + male	283	0	24.7 (14.1) at diagnosis	5-year: 22.3	5-year cumulative incidence of death: 9.8 (95% CI 8.3–11.5)	Inpatient stays: 44 Outpatient stays: 134 ER visits: 60
Pruthi, R.	2016	Retrospective cohort study	UK	Multiple	1997-2009	LN	190	NR	NR	NR	10-year: 88	NR
Feldman, C.H.	2015	Retrospective cohort study	USA	MAX	2000-2006	LN	7 133	90	35 (12.9) at index	NR	NR	Inpatient stays: 1 825
Gomez-Puerta, J.A.	2015	Retrospective cohort study	USA	USRDS	1995-2008	LN + ESRD (Whites)	5 547	78.1	43.3 (16.2) at ESRD onset	NR	1-year survival: 87 5-year survival: 67 10-year survival: 57	NR
						LN + ESRD (African Americans)	6 181	84.6	39.0 (13.4) at ESRD onset	NR	1-year survival: 88 5-year survival: 63 10-year survival: 49	NR
						LN + ESRD (Asians)	683	83.3	36.6 (13.3) at ESRD onset	NR	1-year survival: 95 5-year survival: 81 10-year survival: 67	NR
						LN + ESRD (Native-Americans)	122	79.5	39.0 (14.0) at ESRD onset	NR	1-year survival: 89 5-year survival: 66 10-year survival: 46	NR
Sexton, D.J.	2015	Retrospective cohort study	USA	Single	1995-2010	LN + ESRD	16 650	81.7	<20 years: 6.7 20-29 years: 22.8 30-44 years: 35.1 45-64 years: 27.9 ≥65 years: 7.6	5-year: 33	NR	NR
Furst, D.E.	2013	Retrospective cohort study	USA	MCO claims	2003-2008	LN	907	87.9	18-44 (50.4%), 45-64 (43.9%),	NR	NR	Inpatient stays: 438 (48.2%)

Study description										Results		
**Author**	**Year**	**Type of study**	**Country**	**Number of centers**	**Data collection year**	**Study groups**	**Sample size of study group**	**Gender (% females)**	**Mean (SD) age (y)**	**% ESRD**	**Survival/mortality**	**Healthcare utilization**
									65+ (5.7%) At index [a]			Outpatient visits: 904 (99.7%) ER visits: 431 (47.5%)
Rong, R	2022	Retrospective cohort study	China	Single	1996-2011	LN + hypertensive	764	81.2	Mean 33.2 (13.3) at renal biopsy	Median follow-up of 52 months: ~5.1 (39/764)	Survival: 5, 10, 15-year: 83.0, 74.1, 66.5	NR
						LN + normotensive	642	84.9	29.3 (11.3) at renal biopsy	Median follow-up of 52 months: ~5.5 (35/642)	Survival: 5, 10, 15-year: 91.2, 86.3, 81.3	
Sim, J	2018	Retrospective cohort study	USA	Multiple	2000-2011	LN	411	85.6	31.1 (SD 12.8) mean at biopsy	4.5-year: 12	NR	NR
Bell, C	2023	Retrospective cohort study	USA	Optum	2016-2019	LN	2 310	86.2	60.2 (15.4) at diagnosis	NR	NR	Inpatient stays: 966 (41.8%) Outpatient stays: 2 085 (90.3%) ER visits: 1 422 (61.6%) Pharmacy claims: 2 280 (98.7%)

ER, emergency room; ESRD, end-stage renal disease; IQR, inter-quartile range; LN, lupus nephritis; MCO, managed care organization; NR, not reported; SD, standard deviation; USRDS, United States Renal Data System

[a] Mean (SD) not reported

Table S5 Characteristics of studies reporting on the humanistic burden of LN

Study description										Results	
**Author**	**Year**	**Type of study**	**Country**	**Number of centers**	**Data collection year**	**Study groups**	**Sample size of study group**	**Gender (% females)**	**Mean (SD) age (y)**	**HRQoL tools**	**Key results**
Hu, Y.	2022	Case-control study	China	Single	2018-2021	LN	50	86.0	46.3 (16.7) after enrollment	HADS	HADS-A: median 7.0 (IQR: 6.0-10.0) HADS-D: median 7.5 (IQR: 6.0-11.0) Anxiety rate: 38% Depression rate: 50%
Jolly, M.	2018	Cross-sectional study	Global (USA, Canada, Mexico, Argentina, Turkey, The Philippines, China)	LupusPRO	NR	Non-active-LN	410	NR	41.1 (13.1) at time of study	LupusPRO	SLE patients with LN have poor HRQoL, and SLE patients with active LN have poorer HRQoL than those with non-active LN
						Active LN	129		37.9 (12.9) at time of study		
Mozaffarian, N.	2016	Cross-sectional study	USA	Cross-sectional data from US Adelphi Lupus DSP (no of centers unclear)	2009-2010	LN	206	88.8	39.4 (13.1) at time of study	DSP survey data	Dissatisfaction with disease control was reported by 25% of patients and ~33% of nephrologists. Dissatisfaction was associated with LN severity and various signs and symptoms of disease.

DSP, Disease Specific Programme; HADS, Hospital Anxiety and Depression Scale; HADS-A, Hospital Anxiety and Depression Scale-Anxiety; HADS-D, Hospital Anxiety and Depression Scale-Depression; HRQoL, health-related quality of life; IQR, interquartile range; LN, lupus nephritis; NR, not reported; SLE, systemic lupus erythematosus

Table S6 Characteristics of studies reporting on the economic burden of LN

Study description										Results
**Author**	**Year**	**Type of study**	**Country**	**Number of centers/Name of database**	**Data collection year**	**Study groups**	**Sample size of study group**	**Gender (% females)**	**Mean (SD) age (y)**	**Direct costs ($)**
Bell, C.F.	2022	Retrospective cohort study	USA	Optum	2015-2019	LN	2 916	83.5	65 (14.9) at diagnosis	Mean total annual healthcare cost per flare: LN + any severity of a flare: 5 842 LN + mild flare: 2 753 LN + moderate flare: 4 561 LN + severe flare: 29 148
Dall'Era, M.	2023	Retrospective cohort study	USA	Optum	2007-2019	LN	21 251	86.9	60.3 (15.7) at baseline	All-cause monthly medical costs: Low disease activity: 2 523 Active disease: 4 777 End-stage renal disease: 18 084
Furst, D.E.	2013	Retrospective cohort study	USA	MCO claims database	2003-2008	LN	907	87.9	18-44 (50.4%), 45-64 (43.9%), 65+ (5.7%) At index [a]	Mean total healthcare annual cost per patient: 33 472
Bell, C	2023	Retrospective cohort study	USA	Optum	2016-2019	LN	2 310	86.2	60.2 (15.4) at diagnosis	Mean total all-cause annual costs per patient (including costs for inpatient stays and outpatient visits): 50 975

										Mean (SD) total all-cause costs per patient in the LN cohort (50 975 [86 281]) were also significantly higher vs the SLE without LN cohort (26 262 [52 720]), p<0.001.

LN, lupus nephritis; MCO, managed care organization; SD, standard deviation; SLE, systemic lupus erythematosus
[a] Mean (SD) not reported

Table S7 Characteristics of studies reporting on MN epidemiology

Study description										Results		
**Author**	**Year**	**Type of study**	**Country**	**Number of centers**	**Data collection year**	**Study groups**	**Sample size of study group**	**Gender (% females)**	**Mean (SD) age (y)**	**Prevalence**	**Incidence**	**Proportion of anti-PLA2R positive patients (%)**
Hu, R.	2020	Cross-sectional retrospective study	China	1	2009-2018	MN	8 644	43.5	≤14: 2.1%<br>15–19:11.1%<br>20–39: 17.4%<br>40–59: 34.7%<br>≥60: 43% [c]<br>age at biopsy	2009-2013: 15.98%<br>2014-2018: 30.81%<br>P=0.0004	As % of patients with MN:<br>~25<br>(8 644/34 630)	NR
Pruthi, R.	2016	Cross-sectional retrospective study	UK	Multiple	1997-2009	MN	183	NR	NR	NR	As % of patients with MN:<br>~3.9 (183/4 750)	NR
Glenn, D. A	2020	Prospective, multicenter, observational cohort study	US	72	2014-2017	MN	329	NR	≤18: 5%<br>>18: 28%<br>At enrolment [c]	NR	As % of patients with MN: ~19 (329/1,741)	NR
Ronco, P.	2021	Disease review	France & US	NA	NA	MN	NA	NA	Mean age 50-60 years at diagnosis	NR	10-12 per million in North America<br>2-17 per million in Europe	NR
Storrar, J	2022	Retrospective cohort study	UK	1	2000-2019	Primary MN	178	36.5	Median 58.3 (IQR 44.2-67.1) [c] at kidney biopsy	NR	NR	59.6[b]
Yin	2020	Retrospective cohort study	China	1	2015-2018	SAb+/GAg+	81	40.7	47 (10.3) at baseline	NR	NR	71 (81/114)
						SAb–/GAg+	23	52.2	50.2 (15.2) at baseline	NR	NR	NA
						SAb–/GAg–	10	40.0	45.7 (11) at baseline	NR	NR	NA
Deng	2022	Retrospective cohort study	China	1	2017-2021	PLA2Rab non high titer overall	316	29.8	Median 53 (IQR 42-64) at baseline [c]	NR	NR	316/447
						PLA2Rab high titer overall	131	24.5	Median 55 (IQR 47-63) at baseline [c]		NR	131/447
						PLA2Rab non high	204	27.9	Median 53 (IQR 43-64) at baseline [c]		NR	204/275

						titer treated with CTX						
						PLA2Rab high titer treated with CTX	71	28.2	Median 54 (IQR: 47-61) at baseline [c]		NR	71/275
						PLA2Rab nonhigh titer treated with TAC	112	33.0	Median 53 (IQR: 37-64) at baseline [c]		NR	112/172
						PLA2Rab high titer treated with TAC	60	26.7	Median 56 (IQR: 47-65) at baseline [c]		NR	60/172
Sun	2022	Retrospective cohort study	China	NR	2018-2019	Serum anti-PLA2R ≥150	155	24.5	54.1 (11.8) at kidney biopsy	NR	NR	62% are PLA2R+
						Serum anti-PLA2R <150						
						Serum anti-PLA2R–						
Zhang	2019	Retrospective cohort study	China	1	2014-2016	Serum anti-PLA2R+	71	42.3	54.3 (14) at kidney biopsy	NR	NR	80% PLA2R+
						Serum anti-PLA2R–	18	38.9	46.7 (13.3) at kidney biopsy			

CTX, cyclophosphamide; ESRD, end-stage renal disease; HCC, high-cost cohort; IQR, interquartile range; MN, membranous nephropathy; NHCC, non-high-cost cohort; NA, not applicable; NR, not reported; PLA2R, phospholipase A2 receptor; SD, standard deviation; TAC, tacrolimus

[a]End-stage disease included end-stage renal disease, chronic kidney disease stage 4 or 5, renal transplants, presence of an erythropoietin drug, or dialysis.

[b]Anti-PLA2R analysis was not available during the earlier years of the study period and consequently anti-PLA2R results were only available in 57 patients. Of those tested, 59.6% were positive.

[c] Mean (SD) not reported

Table S8 Characteristics of studies reporting on the clinical burden of MN

Study description										Results			
Author	Year	Type of study	Country	Number of centers	Data collection year	Study groups	Sample size of study group	Gender (% females)	Mean (SD) age (y)	% ESRD	Survival/mortality	HC utilization	Remission
Pruthi, R.	2016	Cross-sectional retrospective study	UK	Multiple	1997-2009	MN	183	NR	NR	NR	Survival: 154<br>Deaths:29<br>Median follow-up to death: 6.2 years	NR	NR
Glenn, D. A	2020	Prospective, multicenter, observational cohort study	US	72	2014-2017	MN	329	NR	≤18: 5%<br>>18: 28%<br>At enrolment [c]	NR	NR	Inpatient stays: 25<br>ER visits: 25[a]	NR
Nazareth, T.A	2019	Retrospective observational analysis	US	IBM MarketScan database	2012-2015	MN HCC	134	39.6	44.5 (NR) at diagnosis	NR	NR	Inpatient stays: 1.97<br>Mean inpatient LoS: 14.2 days<br>Outpatient office visits: 5.17<br>Other outpatient visits: 5.91<br>ER visits: 1.67<br>Pharmacy claims: 1.67[b]	NR
						MN NHCC	2 555	40.6	46.5 (NR) at diagnosis			Inpatient stays: 0.31<br>Mean inpatient LoS: 1.4 days<br>Outpatient office visits: 9.39	

												Other outpatient visits: 21.23<br>ER visits: 0.54<br>Pharmacy claims: 0.54[b]	
Storrar, J	2022	Retrospective cohort study	UK	1	2000-2019	Primary MN	178	36.5	Median 58.3 (IQR: 44.2-67.1) [c] at kidney biopsy	NR	NR	NR	Spontaneous remission: 33.7%<br>Complete remission: 37.6%<br>Nephrotic proteinuria progression: 39.9%
Yin	2020	Retrospective cohort study	China	1	2015-2018	12 months follow-up for Sab–/GAg–	10	40.0	45.7 (11.0) at baseline	0	NR	NR	Partial: 10%<br>Complete: 90%
						12 months follow-up for SAb–/GAg+	23	52.2	50.2 (15.2) at baseline	1			Partial: 47.80%<br>Complete: 52.2%
						12 months follow-up for SAb+/GAg+	81	40.7	47.0 (10.3) at baseline	3			Partial: 32.10%<br>Complete: 58%
Deng	2022	Retrospective cohort study	China	1	2017-2021	PLA2Rab nonhigh titer overall	316	29.8	Median 53 (IQR 42-64) at baseline [c]	NR	NR	NR	Partial: 44.8%<br>Complete: 44.7%
						PLA2Rab high titer overall	131	24.5	Median 55 (IQR 47-63) at baseline [c]				Partial: 23.5%<br>Complete: 20%
						PLA2Rab nonhigh titer treated with CTX	204	27.9	Median 53 (IQR 43-64) at baseline [c]				Partial: 44.6%<br>Complete: 45.2%
						PLA2Rab high titer treated with CTX	71	28.2	Median 54 (IQR 47-61) at baseline [c]				Partial: 28.6%<br>Complete: 27%

						PLA2Rab non high titer treated with TAC	112	33.0	Median 53 (IQR: 37-64) at baseline [c]				Partial: 44.9% Complete: 43.9%
						PLA2Rab high titer treated with TAC	60	26.7	Median 56 (IQR: 47-65) at baseline [c]				Partial: 17.3% Complete: 11.5%
Sun	2022	Retrospective cohort study	China	NR	2018-2019	Serum anti-PLA2R+	155	24.5	54.1 (11.8) at kidney biopsy	NR	NR	NR	Partial: 29.5% Complete: 25.6%
						Serum anti-PLA2R-							Partial: 23% Complete: 42.6%
						SAb–/GAg–							Partial: 21.4% Complete: 35.7%
						SAb–/GAg+							Partial: 45.4% Complete: 27.3%
						SAb <150/GAg–							Partial: 42.1% Complete: 26.3%
						SAb <150/GAg–							Partial: 19.4% Complete: 29%
						SAb ≥150/GAg–							Partial: 50% Complete: 50%
						SAb ≥150/GAg+							Partial: 33.3% Complete: 25%
Zhang	2019	Retrospective cohort study	China	1	2014-2016	Serum anti-PLA2R+	71	42.2	54.3 (14.0) at kidney biopsy	NR	NR	NR	Partial: 50% Complete: 37.5%
						Serum anti-PLA2R+ CTX							Partial: 59.1% Complete: 27.3%
						Serum anti-							Partial: 30% Complete: 60%

						PLA2R+ TAC							
						Serum anti-PLA2R–							Partial: 33.3% Complete: 66.7%
						Serum anti-PLA2R– CTX	18	38.9	46.7 (13.3) at kidney biopsy				Partial: 40% Complete: 60%
						Serum anti-PLA2R– TAC							Partial: 0% Complete: 100%

[a]Number of infections. Described as "Risk factors for time to first infection-related acute care event (hospitalization or emergency department visit"; [b]HC utilization data reported as average number of visits; [c]Mean (SD) not reported

CTX, cyclophosphamide; GAg, glomerular PLA2R antigen; HCC, high-cost cohort; IQR, interquartile range; LoS, length of stay; MN, membranous nephropathy; NHCC, non-high-cost cohort; NR, not reported; PLA2R, phospholipase A2 receptor; SAb, serum PLA2R antibody; SD, standard deviation; TAC, tacrolimus

Table S9 Characteristics of studies reporting on the economic burden of MN

Study description										Results
**Author**	**Year**	**Type of study**	**Country**	**Number of centers**	**Data collection year**	**Study groups**	**Sample size of study group**	**Gender (% females)**	**Mean (SD) age (y)**	**Direct costs ($)**
Nazareth, T.A	2019	Retrospective observational analysis	US	IBM MarketScan database	2012-2015	MN HCC	134	39.6	44.5 (NR) at diagnosis	Prescription costs: $103 382<br>Inpatient costs: $111 140<br>Outpatient costs: $187 085<br>Among outpatient costs:<br>Outpatient office: $2 555<br>Outpatient ED: $1 787<br>Outpatient other: $182 744<br>Outpatient hospital: $88 729<br>ESRD facilities: $67 302<br>Other facilities: $26 731
						MN NHCC	2 555	40.6	46.5 (NR) at diagnosis	Prescription costs: $5 050<br>Inpatient costs: $7 761<br>Outpatient costs: $14 343<br>Among outpatient costs:<br>Outpatient office: $1 162<br>Outpatient ED: $498<br>Outpatient other: $12 683<br>Outpatient hospital: $7 149<br>ESRD facilities: $2 629<br>Other facilities: $2 905

ESRD, end-stage renal disease; HCC, high-cost cohort; MN, membranous nephropathy; NHCC, non-high-cost cohort; NR, not reported; SD, standard deviation

Table S10 Variation in definitions of complete and partial response used for patients with MN

Study	Definition of complete response	Definition of partial response
Zhang et al 2019	Proteinuria <0.3 g/day, and normal serum albumin and creatinine levels	Proteinuria <3.5 g/day and a 50% reduction in baseline levels, with improving serum albumin and creatinine
Sun et al 2022	Urinary protein excretion <0.5 g/day (uPCR <500 mg/g)	≥50% reduction from peak values of urinary protein and urinary protein excretion <3.5 g/day
Yin et al 2020	24-h urinary protein <0.3 g or urinary albumin/creatinine <0.3 g/g and serum albumin >40 g/L	24-h urinary protein of 0.3-3.5 g or urinary albumin/creatinine of 0.3-3.5 g/g and serum albumin >30 g/L
Storrar J et al 2022	Complete remission was defined as return to baseline eGFR and uPCR <25 g/mol	Return to baseline eGFR and uPCR <350 g/mol with ≥50% reduction from peak value
Deng et al 2022	Urinary protein <0.3 g/24 h, accompanied by normal serum albumin and serum creatinine	Urinary protein <0.3 g/24 h, and ≥50% reduction from accompanied by an improvement or normalization of the serum albumin and stable serum creatinine

eGFR, estimated glomerular filtration rate; MN, membranous nephropathy; uPCR, urine protein creatinine ratio
